# The effect of lipoxin A4 on E. coli LPS-induced osteoclastogenesis

**DOI:** 10.1007/s00784-020-03385-3

**Published:** 2020-06-06

**Authors:** Muhanad Ali, Nathan Kucko, John A. Jansen, Fang Yang, X. Frank Walboomers

**Affiliations:** Department of Dentistry, Biomaterials, Radboud Institute for Molecular Life Sciences, Radboud University Medical Center, Philips van Leydenlaan 25, 6525EX Nijmegen, The Netherlands

**Keywords:** Osteoclast, Inflammation, Bone resorption, Lipoxin A4, Periodontitis

## Abstract

**Objectives:**

The objective of the present study was to investigate the effect of lipoxin-type A4 (LXA4) on bacterial-induced osteoclastogenesis.

**Material and methods:**

Human periodontal ligament cells (PDLCs) in coculture with osteoclast precursors (RAW264.7 cells) were exposed to bacterial stimulation with lipopolysaccharide (LPS) to induce inflammation. After 24 h, cells were treated to 100 ng/ml of LXA4 and 50 ng/ml of forymul peptide receptor 2 (FPR2/ALX) receptor antagonist (Boc-2). After 5 days, osteoclastic resorptive activity was assessed on calcium phosphate (CaP) synthetic bone substitute. Additionally, osteoclastic differentiation was evaluated using tartrate-resistant acid phosphatase (TRAP) staining, TRAP enzymatic activity assay, and on the expression of osteoclast-specific genes.

**Results:**

We found that stimulation of in the osteoclasts with LPS-stimulated PDLCs induced a significant increase in tartrate-resistant acid phosphatase (TRAP) positive cells, higher resorptive activity, and enhanced expression of specific genes. Meanwhile, LXA4-treatment exhibited strong anti-inflammatory activity, and was able to reverse these inflammatory effects.

**Conclusions:**

We conclude that (1) PDLCs are a potential target for treating bacterial-induced bone resorption in patients with periodontal disease, and (2) LXA4 is a suitable candidate for such therapy.

**Clinical relevance:**

The results prove that lipoxins have a protective role in bacterial-induced periodontal inflammation and alveolar bone resorption, which can be translated into a clinical beneficial alterative treatment.

## Introduction

Periodontal disease, the most common chronic disease in humans, is characterized by inflammation of the supporting tissues around the teeth [1]. Alveolar bone loss is one of the major hallmarks for disease progression, and combatting bone loss is therefore key to treating periodontal disease [2]. Normal bone remodeling is a dynamic process maintained by a balance between bone formation and resorption [3, 4]. In periodontal disease, chronic inflammation disrupts the homeostatic balance between bone formation and bone resorption, in favor of bone loss [5]. There is evidence that the periodontal ligament (PDL) plays an important role in the remodeling of alveolar bone [5].

Periodontal ligament cells (PDLCs) are specialized spindle-shaped cells responsible for maintaining the integrity of the ligament that is connecting the tooth root cementum to the alveolar jaw bone [6]. However, in chronic inflammation (i.e., periodontitis), the phenotype of PDLCs changes, leading to a tissue-destructive response [7]. Microorganisms (e.g., *Porphyromonas gingivalis*, *Fusobacterium nucleatum*, and *Escherichia coli*) promote the PDLCs to interact with osteoclast progenitor cells (OPCs), which leads to the differentiation of these OPCs into mature osteoclasts [8, 9]. During this interaction, PDLCs attract and stimulate the differentiation of OPCs towards mature osteoclasts via expression of adhesion molecules, such as adhesion molecule-1 (ICAM-1), and expression of osteoclastogenesis stimulatory factors, such as RANKL, M-CSF, and tumor necrosis factor-alpha (TNFα) [10]. In vivo, this leads to retraction of PDLCs and migration of OPCs to the bone surface; where the OPCs mature into bone-resorbing osteoclasts [11–14]. Consequently, the cascade of infection, inflammation mediated by the PDLCs, and osteoclast differentiation presents an important drug target for therapeutic agents aimed at suppressing bone loss.

Over the last decade, there has been an increasing number of studies on the anti-inflammatory effect of specialized pro-resolving mediators (SPMs), and especially of lipoxins (LXs) [15, 16]. More recently, other effects of LXs have been observed, including the role of LXs in preventing bone resorption [17]. LXs are endogenous metabolic products derived from arachidonic acid metabolism, which have important pro-resolving and anti-inflammatory properties [18–20]. Lipoxin-type A4 (LXA4) binds specifically to a G protein-coupled N-formyl peptide receptor 2 (FPR2/ALXR) expressed by a variety of inflammatory cells, and which is also expressed in PDLCs [21]. The binding of LXA4 to its receptor induces a pro-resolving effect mainly by suppressing the expression of inflammatory mediators (e.g., IL-1β and IL-6) via inhibition of multiple signaling pathways, including receptor activator of nuclear factor-κB (NF-κB), which opposes inflammation [17, 22, 23]. The anti-inflammatory effect of LXA4 is well documented in the literature [22, 24]. However, the effect of LXA4 on bacterial-induced osteoclastic differentiation during cell-cell contact between PDLCs and OPCs remains elusive.

Given the relationship between inflammation and osteoclast formation and function, we hypothesized that (1) LXA4 would have an inhibitory effect on bacterial-induced osteoclastogenesis, and (2) this effect would be reversed by FPR2/ALXR antagonist, Boc-2. To test this hypothesis, we developed a coculture model derived from a murine RAW264.7 osteoclast cell line, in direct contact with primary human PDLCs in vitro. After induction of an inflammatory phenotype of the PDLC (by the addition of *E. coli* lipopolysaccharide; LPS), the osteoclastic differentiation was evaluated by means of TRAP staining, TRAP enzymatic activity assay, resorption of a calcium phosphate substrate, and expression levels of osteoclast-specific genes.

## Material and methods

### Reagents and chemicals

Synthetic LXA4 was purchased from Cayman Chemical (Ann Arbor, MI, USA), and Boc-Phe-Leu-Phe-Leu-Phe (Boc-2) was purchased from Phoenix Pharmaceutical (Burlingame, CA, USA). Dulbecco’s Modified Eagle’s Medium (DMEM/F-12), Minimum Essential Medium Eagle alpha (αMEM), sterile phosphate buffered saline (PBS), fetal bovine serum (FBS), penicillin-streptomycin (PS), and trypsin-EDTA solution were all purchased from Gibco (Delft, Netherlands). Phosphate buffered saline (PBS) tablets and Acid Phosphatase Leukocyte Staining Kit (387A) were purchased from Sigma-Aldrich (St. Louis, MO, USA). Commercially available preparations of LPS from *E. Coli* were purchased from InvivoGen (San Diego, CA, USA). Murine activator of nuclear factor-κ B ligand (mRANKL) was purchased from Peprotech (Rocky Hill, NJ, USA). All cell culture flasks and plates were purchased from Greiner Bio-one (Frickenhausen, Germany). The RNA Isolation Kit was obtained from Qiagen (Venlo, Netherlands). TaqMan Reverse Transcription kit and Fast SYBR Green Master Mix Kit were obtained from Thermo Fisher Scientific (Breda, Netherlands).

### Calcium phosphate coating

A biomimetic calcium phosphate (CaP) coating was applied to glass coverslips using a two-stage process as described elsewhere [25, 26]. The first stage involved immersing the glass slides in a 2.5-fold simulated body fluid (SBF) solution (1 ml/well) at room temperature under light agitation for 3 days and daily refreshment of the solution. A total of 2.5-fold SBF was prepared by mixing Tris buffer (0.05 M; pH adjusted to 7.4 using 1 M HCl), calcium stock solution, and a phosphate stock solution together in a ratio of 2:1:1. The calcium and phosphate stock solution was prepared by dissolving the reagents specified in Table 1 in Tris buffer solution. The second stage involved immersing the glass coverslips in a calcium phosphate solution (CPS) (1 ml/well) for 1 day at room temperature under light agitation. CPS was prepared by fully dissolving each reagent in the order listed in Table 1 in MilliQ water, while ensuring that the pH did not rise above 8 using 1 M HCl. Once all the Tris was fully dissolved, the pH was adjusted to 7.4. After 1 day of soaking, the CPS was aspirated from the wells and 70% ethanol was added and left to evaporate to sterilize the CaP-coated glass coverslips. Lastly, the coverslips were gently rinsed three times with MilliQ water and dried overnight at room temperature before use in cell culture. Prior to cell seeding, the CaP-coated glass coverslips were incubated in FBS for 1 h at 37 °C.

**Table 1:** Reagents used to prepare calcium and phosphate stock solutions, as well as CPS. Reagents for the calcium and phosphate stock solutions were dissolved using a 0.05 M Tris buffer solution with a pH adjusted to 7.4 using 1 M HCl, while reagents for the CPS were dissolved in MilliQ water

Solution	Reagent formula	Molar mass	Molarity (M)
Calcium stock	CaCl_2_ ● 2H_2_O	147.02	0.025
	NaCl	58.44	1.37
	MgCl_2_ ● 6H_2_O	203.30	0.015
Phosphate stock	NaH_2_PO_4_ ● 2H_2_O	156.01	0.0111
	NaHCO_3_	84.01	0.042
CPS	NaH_2_PO_4_ ● 2H_2_O	156.01	0.00225
	CaCl_2_ ● 2H_2_O	147.02	0.004
	NaCl	58.44	0.14
	Tris	121.1356	0.05

#### Coating characterization

##### SEM

To evaluate the morphology of CaP-coated glass coverslips with 12-mm diameter (VWR B.V., Amsterdam, The Netherlands), specimens were sputter-coated with a 30-nm thin chromium layer and imaged using a scanning electron microscope (SEM; Zeiss Sigma 300, Zeiss AG, Oberkochen, Germany).

##### X-ray diffraction

A PANalytical X’Pert^3^ X-ray diffractometer (Malvern Panalytical B.V., Eindhoven, The Netherlands) with a Cu Kα radiation source was employed to perform thin film X-ray diffraction (XRD) on CaP-coated glass slides using an incidence grazing angle of 2.5°. The measurements were performed at a voltage and current of 45 kV and 40 mA, respectively, with a step size of 0.02° and a count time of 1 s per step. All scans were performed between 10° and 50° 2*θ*.

##### Fourier transform infrared spectral study spectroscopy

Characterization of the crystal phase of the calcium phosphate coated on glass slides was investigated using FT-IR spectroscopy (Spectrum Two FT-IR Spectrometer, PerkinElmer, Waltham, USA). The samples were scanned in the 4000–650 cm^−1^ range with an ATR accessory.

##### Surface profile

Characterization of the surface profile of the calcium phosphate coated on glass slides was investigated using Universal Surface Tester (UST®, Innowep GmbH, Wuerzburg, Germany). The samples were secured to the sample stage and measurements were taken using the stylus diamond tip (*R* = 2 μm, 60°) connected to the measurement head with velocity of 0.1 mm/s bearing the load of 1.0 mN at increments of 2.0 μm for a total distance of 5 mm.

### Cell culture

All experiments were done in accordance with the national guidelines for working with human materials (Dutch Federation of biomedical scientific societies, human tissue, and medical research; code of conduct for responsible use. Available at https://www.federa.org/). The RAW 264.7 murine macrophage cell line (ATCC®TIB-71™) was purchased from the American Type Culture Collection (Manassas, VA, USA) and maintained in αMEM medium supplemented with 10% FBS and 1% PS at 37 °C in a humidified atmosphere of 5% CO_2_ until 80% confluence (number of passages was < 10). After informed patient consent, human periodontal ligament cells (PDLCs) were harvested from several healthy adult donors who underwent extraction of a third molar as previously described [27]. PDLCs were cultured at 37 °C in a humidified atmosphere of 95% air and 5% CO_2_. The medium was replaced every 2 to 3 days until 50% confluence was reached, and then, the PDLCs were passaged before being frozen in medium supplemented with 10% dimethyl sulfoxide (DMSO; Sigma-Aldrich). After defrosting, PDLCs were passaged again and maintained in sterile 75 cm^2^ culture flasks with DMEM medium supplemented with 10% FBS and 1% PS and cultured at 37 °C in a humidified atmosphere of 95% air and 5% CO_2_. For all experiments, the cell passage was ≤ 5.

#### RANKL-induced osteoclasts

As a positive test for osteoclast differentiation, RAW264.7 cells were seeded in a 24-well plate (2 × 10^3^ cells/cm^3^) and cultured in αMEM supplemented with 10% FBS and 1% PS. At day 1, medium was refreshed with differentiation medium (50 ng/ml of murine RANKL) to induce osteoclast formation. At day 2, medium was refreshed with differentiation medium supplemented with LXA4 100 ng/ml and Boc-2 50 ng/ml, and then, cells were returned to the incubator at 37 °C in a humidified atmosphere of 95% air and 5% CO_2._ The medium was replaced every 2 days. After 5 days, the cells and medium were collected for TRAP staining and enzymatic assays. Parallel samples were collected for mRNA extraction.

#### LPS/PDLC-induced osteoclasts

**Fig. 1:**
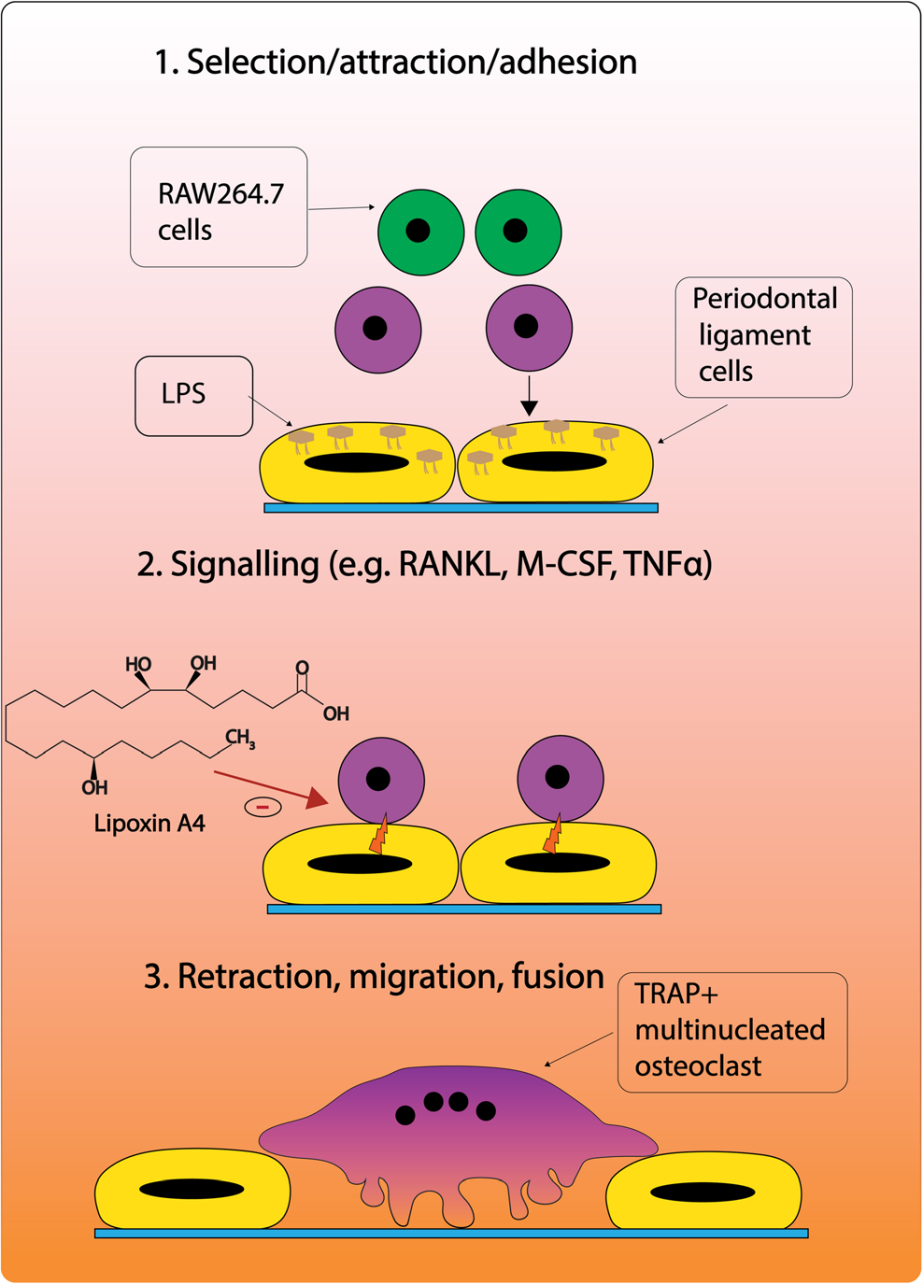
Sequence of events in periodontal ligament cells/lipopolysaccharide (LPS/PDLC)-induced osteoclastic differentiation of RAW264.7. 1. LPS-stimulated PDLCs select and attract osteoclast precursors (e.g., RAW264.7). 2. Following adhesion of RAW246.7 cells, PDLCs increase the expression of osteoclast promoting signaling molecules, such as RANKL, M-CSF, and TNFα. 3. PDLCs retract leading to the maturation and migration of RAW246.7 to the bone surface. The following sequence of events and illustration is modified from Sokos et al. [5]. Receptor activator of nuclear factor-κB (RANKL), macrophage colony-stimulating factor (M-CSF), tumor necrosis factor-alpha (TNFα)

Protocol for direct coculture of human PDLCs and murine RAW264.7 was adapted from a previous protocol [9]. PDLCs were cultured in a 24-well plate (1 × 10^4^ cells/cm^3^) with DMEM medium supplemented with 10% FBS and 1% PS and cultured at 37 °C in a humidified atmosphere of 95% air and 5% CO_2_. After 48 h, RAW 264.7 (2 × 10^3^ cells/cm^3^) were seeded on top of PDLCs using a 1:1 mixture of basic medium (DMEM:αMEM). After 24 h, cell culture medium was refreshed with basic medium supplemented with LPS (1 μg/ml) to let PDLCs induce osteoclast formation in RAW264.7 cells, as shown in Fig. 1. After 24 h, medium was replenished with fresh medium supplemented with LXA4 (100 ng/ml) and Boc-2 (50 ng/ml), and cells were cultured accordingly as before. After 5 days, the cells and medium were collected for TRAP staining and enzymatic assays. Parallel samples were collected for mRNA extraction.

#### Osteoclast purification and transfer

Purification and transfer of osteoclasts was adapted from Maria et al. (2014) [26]. After induction of osteoclasts, both by RANKL and by LPS/PDLC induction, the medium was aspirated and cells were washed twice with pre-warmed sterile PBS. To detach undifferentiated cells, 0.25% trypsin (1 ml/well) was added and the plate was returned to the incubator at 37 °C for 1 min. The medium containing undifferenced mononucleated cells was aspirated, leaving behind large multinucleated cells (> 100 μm in diameter). The differentiated osteoclasts were dislodged from the plate by gently pipetting the solution, while observing under the microscope. The differentiated osteoclasts were then seeded at the exact seeding density on top of calcium phosphate-coated glass slides that had been incubated prior with FBS for 1 h at 37 °C. Tissue culture plates were returned to the incubator carefully and maintained for 48 h at 37 °C in a humidified atmosphere of 95% air and 5% CO_2_.

### Measurements

#### Osteoclast resorption

After incubation of osteoclast on CaP-coated glass slides for 48 h, cell culture medium was aspirated, and cells were washed twice with sterile PBS. The cells were then removed by incubating with sodium chloride (1 M) in 0.2% Triton-X-100 for 1–2 min, and subsequently washed with MilliQ water. To stain for calcium, the CaP-coated glass slides were incubated in silver nitrate solution (5% AgNO_3_) at RT under an UV lamp for 1 h. Then, the glass slides were washed with distilled water and the coating surface was examined by a light microscope. The osteoclast resorption pits were measured using Fiji 1.51n software (National Institute of Health, Bethesda, MD, USA). To study the morphology of osteoclasts cultured on CaP-coated glass slides, parallel samples containing cells were fixed with 2.5% glutaraldehyde overnight. After fixation, cells were dehydrated in a graded ethanol series: 25, 50, 60, 70, 80, 90, 95, 100%—each for 5 min. The cells were further dehydrated with a graded hexamethyldisilazane (HMDS; Sigma-Aldrich) series diluted in 100% ethanol: 1:2, 1:1, 2:1 (v/v ratios of HMDS; 100% ethanol). Specimens were then treated with undiluted 100% HMDS each for 10 min and allowed to evaporate under the flow hood overnight. Finally, samples were sputter-coated with gold and imaged accordingly by SEM.

#### TRAP staining and TRAP enzymatic activity assay

Tartrate-resistant acid phosphatase (TRAP) enzymatic activity was measured using the Acid Phosphatase Leukocyte Staining Kit (Sigma-Aldrich St. Louis, MO) in accordance with the manufacturer’s instructions. Briefly, 10 ml of acid phosphatase staining solution consisting of 9 ml of pre-warmed MilliQ, 400 μl of acetate solution, 100 μl of naphthol AS-BI phosphoric acid, 200 μl of tartrate solution, and 200 μl diazotized Fast Garnet GBC was prepared. To stain for TRAP activity, cells were rinsed once with PBS before adding 3.7% paraformaldehyde for 5 min at RT. The cells were then rinsed once with deionized water, and staining solution was added for 1 h at 37 °C. After incubation, cells were rinsed again with deionized water, and TRAP-positive multinucleated osteoclasts were observed and photographed using a light microscope (Leica, Germany). TRAP enzymatic activity in the cultured medium was analyzed with a microplate reader (Synergy HTX multi-mode Reader, BioTek, Winooski, VT, USA) at 405 nm.

### RNA extraction and real-time qPCR

RNA from cultured cells was isolated using the RNeasy Mini Kit (Qiagen) according to the manufacturer’s instructions. Briefly, cells were washed with PBS and then lysed with 2-mercaptoethanol (Sigma-Aldrich, St. Louis, USA). The cell extract was collected, mixed with 70% ethanol before isolating RNA using a RNeasy column. Subsequently, the obtained RNA was dissolved in RNase-free water, and the RNA concentration was measured with a spectrophotometer (NanoDrop 2000, Thermo Scientific, Wilmington, DE, USA). RNA was used to reverse transcribe first-strand cDNA using the iScriptTM Select cDNA Synthesis Kit (Bio-Rad, CA, USA). Thereafter, cDNA was further amplified, and the expression of specific genes was quantified using quantitative polymerase chain reaction (qPCR) MasterMix Plus for SYBR® Green I (Eurogentec, Seraing, Belgium) and a real-time PCR detection system (CFX96TM Real-Time PCR Detection System, Bio-Rad). Osteoclastogenic differentiation-related marker genes were evaluated, including tartrate-resistant acid phosphatase (TRAP), receptor activator of nuclear factor kappa-B ligand (RANKL), cathepsin k (CK). The sequence of applied primers is given in Table 2. The expression levels were analyzed and normalized with the house-keeping gene glyceraldehyde 3-phosphate dehydrogenase (GAPDH) by calculating ΔCt (Ct_gene of interest_ - Ct_GAPDH_), and the expression of the tested gene was calculated using the 2^−(ΔCt)^.

**Table 2:** List of primers (sequence 5′ to 3′, sense and antisense).

Primer	Forward 5′-3′	Reverse 5′-3′
RANK	CAGGAGAGGCATTATGAGCA	GGTACTTTCCTGGTTCGCAT
TRAP	GATGCCAGCGACAAGAGGTT	CATACCAGGGGATGTTGCGAA-
CK	GAAGAAGACTCACCAGAAGCAG	TCCAGGTTATGGGCAGAGATT-
GAPDH	TGA CCA CAG TCC ATG CCA TC	GAC GGA CAC ATT GGG GGTA G

### Statistical analysis

Statistical differences were evaluated using one-way analysis of variance (ANOVA) test with a pairwise multiple-comparison test (Tukey test) to assess significant difference in TRAP enzymatic activity, resorption pit formation, and gene expression between groups. All statistical analysis was performed using the GraphPad Prism v.5 (GraphPad Software, San Diego, USA). Data are expressed as mean ± standard deviation (SD). Differences were statistically significant when *p* < 0.05. For all assays, three independent experiments were performed using different cell batches, in which the experimental specimens were present in triplicate (*n* = 3).

## Results

### Characterization of calcium coating

**Fig. 2:**
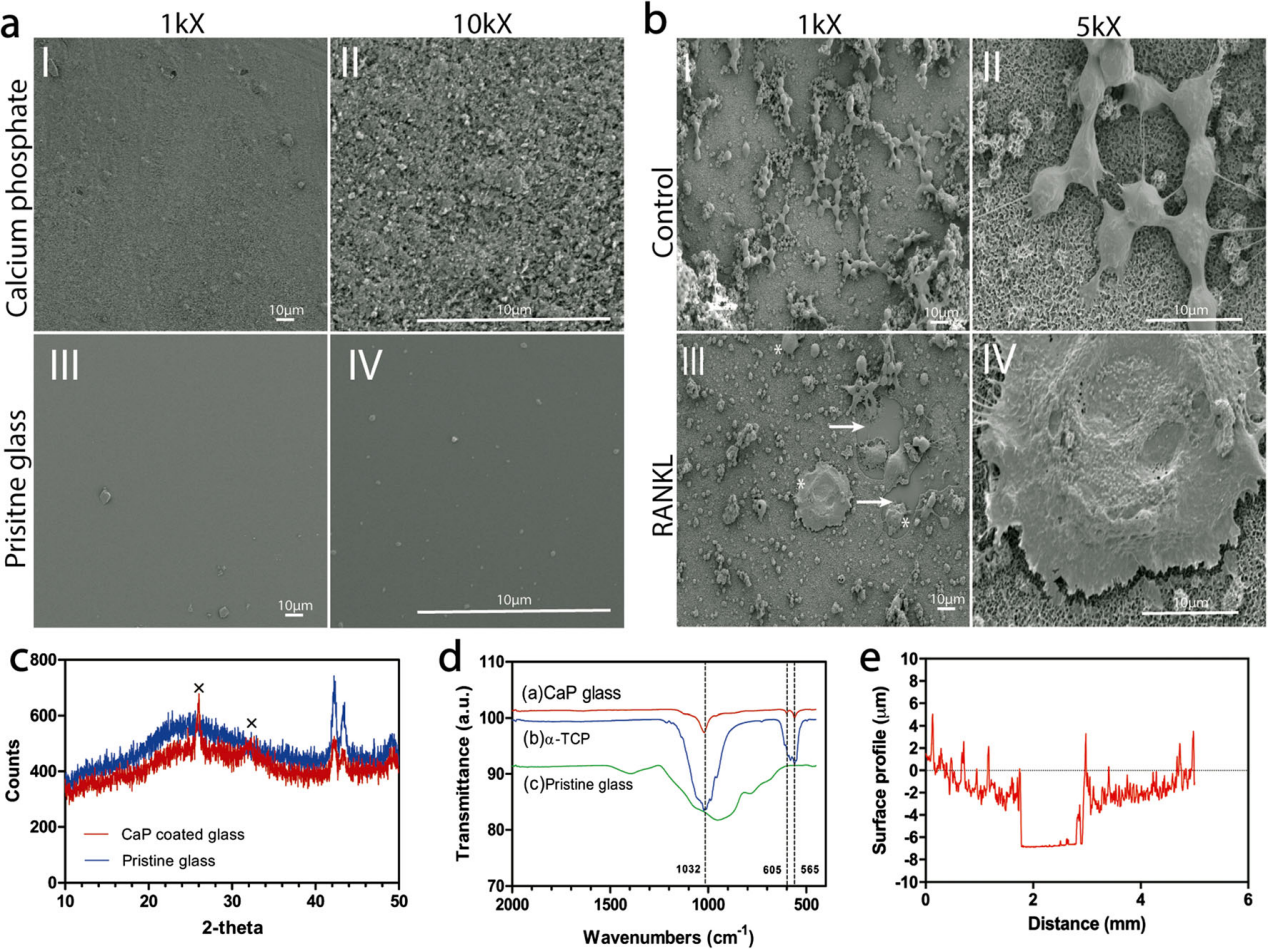
Scanning electron microscopy of (**a**) (I and II) calcium phosphate (CaP) coated glass, and (III and IV) pristine glass as reference. Magnification: × 1000 and × 10,000. Scale bar: 10 μM. (**b**) (I and II) SEM microscopy of RAW246.7 cultured on CaP-coated glass slides cultured with treated with basic medium, or (III and IV) RAW cells cultured in differentiation medium (RANKL) for up to 5 days. Magnification × 1000 and × 5000. Scale bar: 10 μm. Arrows indicate resorption of CaP substrate; asterisks (*) indicate presence of osteoclasts. (**c**) X-ray diffraction (XRD) patterns of the calcium coating on a glass slide. Crosses “×” indicate hydroxyapatite peaks (**d**) FT-IR spectra of (a) CaP glass slide, (b) α-TCP, and (c) pristine glass. Dashed lines indicate phosphate bands. (**e**) Surface characterization of CaP substrate displaying a coating thickness of ~ 5 μm

SEM micrographs of the CaP-coated glass slides (Fig. 2a) showed a homogeneous and uniform distribution, with a coating thickness of ~ 5 μm as detected by a Universal Surface Tester (Fig. 2e). The XRD patterns (Fig. 2c) displayed diffraction lines characteristic of hydroxyapatite (HA), which were comparable with literature [26]. Moreover, FT-IR spectra (Fig. 2d) of the CaP coating revealed characteristic bands at 1032, 605, and 565 cm^−1^, which are attributed to the phosphate groups (PO_4_^3^) in the HA chemical structure [28]. Also, the SEM micrographs revealed that treatment of RAW264.7 cells in just basic medium did not affect the HA coating and left the coating intact (Fig. 2b I and II), while the sheer size and number of osteoclasts (± 50−100 μm) were strongly upregulated upon treatment of RAW246.7 cells with differentiation medium (basic medium supplemented with RANKL) for up to 5 days (Fig. 2B III and IV).

### Osteoclast resorption

**Fig. 3:**
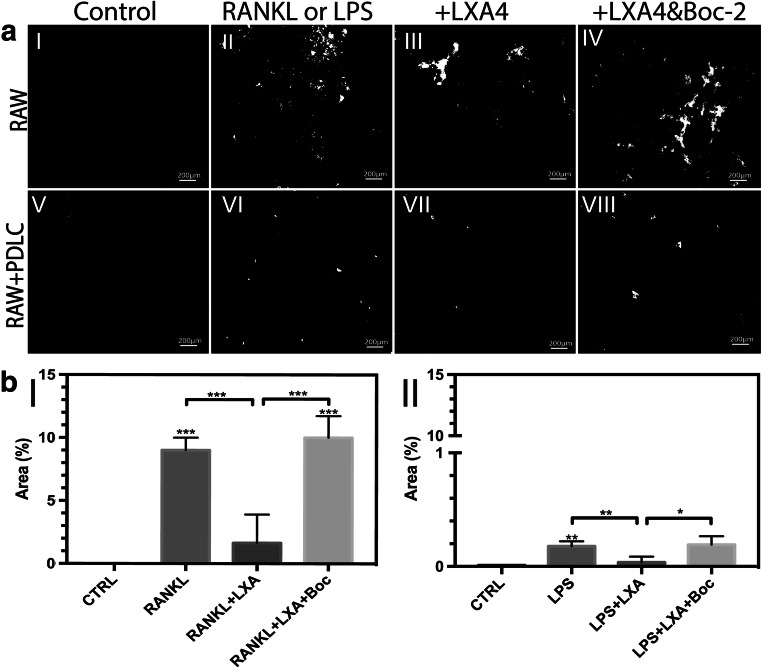
(**a**) Images of calcium phosphate (CaP)-coated glass substrates after staining with silver nitrate (AgNO_3_) following incubation with (I) basic medium (control), (II) RANKL (alone, 50 ng/ml), (III) RANKL+LXA4 (50 ng/ml), (IV) RANKL+LXA4+Boc-2 (10 μM), or in RAW/PDLC coculture in medium supplemented with (V) basic medium (control), (VI) LPS (alone, 10 μg/ml), and (VII) LPS+LXA4 (50 ng/ml), (VIII) LPS+LXA4+Boc-2 (10 μM). Resorbed pits are shown in white, and intact CaP coating is stained black. Note that LXA4 inhibits RANKL or LPS/PDLC-induced osteoclast resorption of CaP coating, and Boc-2 reversed this effect. Magnification: × 10,000. Scale bar: 200 μm. (**b**) Quantification of resorption pit area (%) resorbed by osteoclasts obtained following incubation of RAW264.7 with (I) RANKL (alone), and (II) LPS (alone) under the same conditions. Statistical analysis was performed by one-way ANOVA with Tukey post-test, *n* = 100 *p ≤ 0.05, **p ≤ 0.01, ***p ≤ 0.001. Asterisks on top of the columns indicate significant differences from the control

Microscopic images showed an intact coating in response to cells cultured with medium alone (control) (Fig. 3aI). However, several resorption pits were found on the CaP coating following incubation of osteoclasts with RANKL or with LPS/PDLC stimulation (Fig. 3a II and VI). For both types of stimulation of the RAW cells, the relative area of osteoclasts was visually downregulated in response to LXA4 treatment (Fig. 3a III and VII). Conversely, the inhibitory effect of LXA4 was visibly reversed upon treatment with LXA4 together with its receptor inhibitor, Boc-2 (Fig. 3b IV and VIII).

Furthermore, the total resorbed area (%) relative to the control (medium alone) in response to the indicated condition was measured (Fig. 3b I and II). In agreement with the visual inspection, quantitative analysis revealed no significant changes in resorption activity of the RAW cells supplemented with control medium. However, a significant increase in the resorptive capacity was observed in response to either RANKL or the LPS/PDLC challenge alone compared with control (*p* < 0.001 and *p* < 0.01, respectively). Furthermore, a significant decrease in osteoclastic resorptive activity was measured in response to treatment of both groups with LXA4 compared with RANKL or LPS/PDLC-treatment (*p* < 0.001 and *p* < 0.01, respectively). Conversely, a significant increase in osteoclast resorption activity was observed upon treatment of RANKL or LPS/PDLC-challenged cells with LXA4 and Boc-2, as compared with LXA4 treatment alone (*p* < 0.001 and < 0.05, respectively).

### TRAP staining and TRAP enzymatic activity assay

**Fig. 4:**
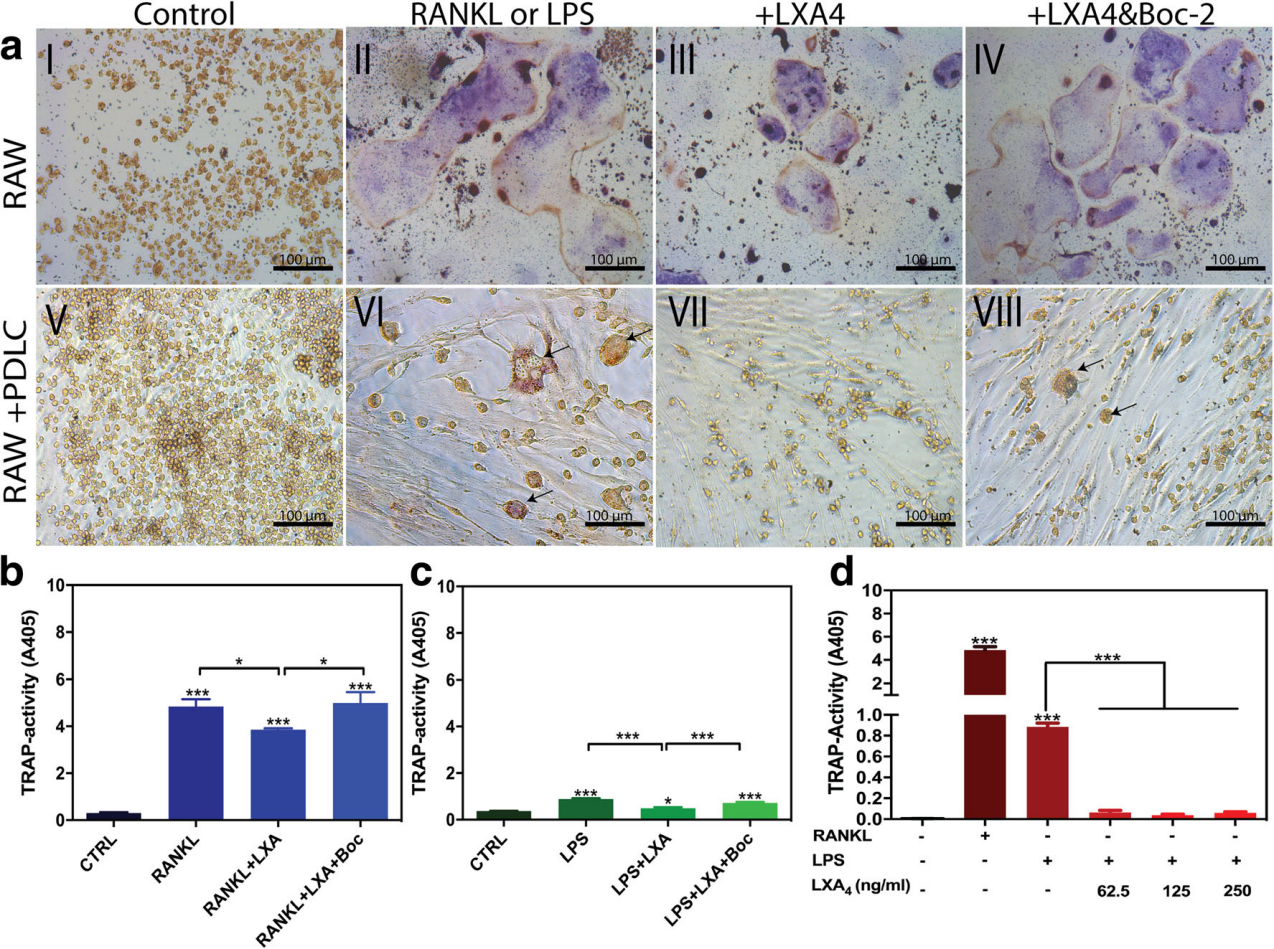
(**a**) Tartrate-resistant acid phosphatase **(**TRAP) staining in RAW264.7 cells in monoculture (I–IV) treated with **(**I) basic medium (control), (II) RANKL (alone, 50 ng/ml), (III) RANKL + LXA4 50 ng/ml, (IV) RANKL+LXA+Boc-2. TRAP staining in RAW264.7 cells in coculture with PDLCs (V–VIII) treated with (V) basic medium (control), (VI) LPS (alone,10 μg/ml), (VII) LPS + LXA4 (50 ng/ml), and (VIII) LPS+LXA+Boc-2 (10 μM). Note that LXA4 inhibits RANKL and LPS/PDLC-induced osteoclastic differentiation, while Boc-2 reversed this response. Magnification: × 20,000. Scale bar: 100 μm. (**b**–**d**) TRAP enzymatic activity in the culture medium obtained from (**b**) RAW264.7 cells in monoculture, and (**c**) RAW264.7 cells in coculture with PDLCs. Note that LXA4 inhibited RANKL, and LPS/PDLC increased TRAP activity. (**d**) Effect of increasing concentration of LXA4 (62.5, 125, and 250 ng/ml) in LPS (50 ng/ml)-stimulated RAW264.7 cells in coculture with PDLCs. Note that all concentrations of LXA4 significantly inhibited TRAP activity. Statistical analysis was performed by one-way ANOVA with Tukey post-test, *n* = 100 *p ≤ 0.05, **p ≤ 0.01, ***p ≤ 0.001. Asterisks on top of the columns indicate significant differences from the control

TRAP staining was used as a marker of osteoclastic differentiation, and visual inspection showed that the number of multinucleated TRAP-positive cells was markedly upregulated in response to stimulation with RANKL or LPS/PDLC (Fig. 4a II and VI, respectively). Characteristically, RANKL-derived osteoclasts were noticeably larger compared with LPS/PDLC-derived osteoclasts. Nevertheless, treatment of RANKL or LPS/PDLC-stimulated osteoclasts with LXA4 significantly downregulated the size and number of osteoclasts (Fig. 4a III and VII). Conversely, treatment with LXA4 along with Boc-2 significantly reversed the inhibitory activity of LXA4 (Fig. 4a IV and VIII).

Furthermore, TRAP activity in cell culture media was also measured to confirm the visual inspections (Fig. 4b**)**. In agreement with morphological assessment, a significant increase in TRAP activity was observed following treatment of RAW264.7 cells with RANKL or LPS/PDLC (*p* < 0.0001 and *p* < 0.001, respectively). Moreover, TRAP activity was significantly decreased back to baseline level upon treatment of RANKL as well as LPS/PDLC-stimulated cells with LXA4 alone (*p* < 0.05 and *p* < 0.001, respectively). Conversely, the inhibitory activity of LXA4 was suppressed by treatment with Boc-2, and TRAP activity was once again significantly different compared with the control (*p* < 0.05 and *p* < 0.001, respectively). No dose-dependent inhibition was observed, as all the LXA4 concentrations investigated strongly inhibited RANKL as well as PDLCs/LPS-induced osteoclastic differentiation (Fig. 4d).

### Real-time PCR

**Fig. 5:**
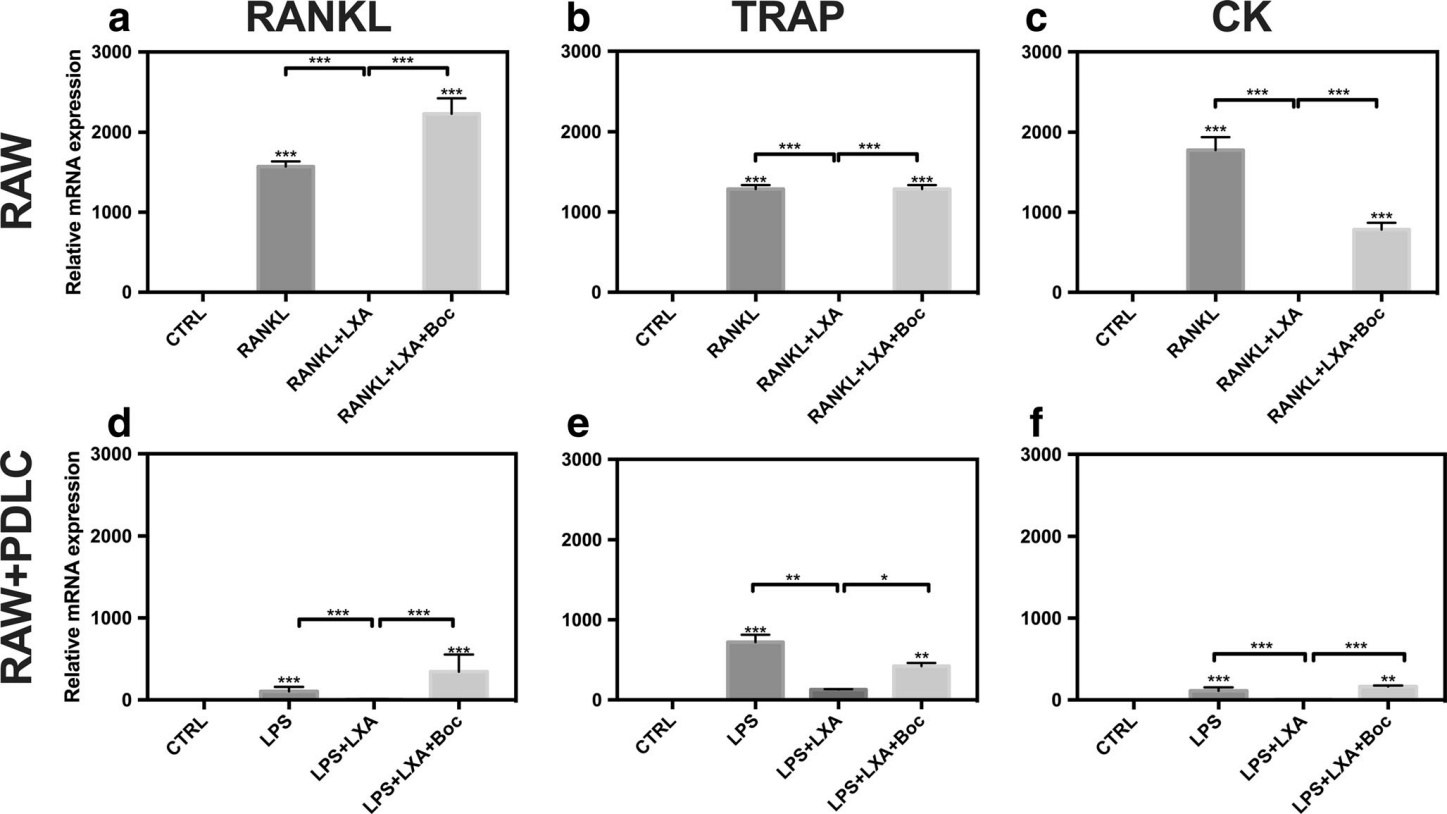
Expression levels of RANKL, TRAP, and CK were determined by qRT-PCR in (**a**–**c**) RANKL-stimulated RAW264.7 monoculture, and (**d**–**f**) LPS/PDLC-stimulated RAW264.7. Note that LXA4 inhibited RANKL and LPS/PDLC-induced expression of osteoclast marker genes, while Boc-2 reversed this response. Expression levels were calculated in relation to internal controls (GAPDH mRNA) with a comparative Ct method. Statistical analysis was performed by one-way ANOVA with Tukey post-test, *n* = 100 *p ≤ 0.05, **p ≤ 0.01, ***p ≤ 0.001. Asterisks on top of the columns indicate significant differences from the control

Finally, the expression of osteoclast-specific genes was analyzed to investigate the underlying mechanism by which LXA4 induces the inhibition of RANKL and LPS/PDLC-stimulated osteoclastogenesis. RANKL stimulation significantly upregulated the expression level of osteoclastogenesis-associated gene expression (RANKL, TRAP, and CK mRNA) in RAW264.7 in monoculture (Fig. 5a–c). Similarly, LPS/PDLC stimulation significantly upregulated the expression levels of osteoclast-specific genes relative to the control (Fig. 5d–f). Accordingly, the expression level of osteoclast genes was markedly higher in response to RANKL treatment compared with LPS/PDLC stimulation. Overall, the treatment of cells with LXA4 significantly downregulated the expression level of osteoclastogenesis marker genes in either case. Lastly, treatment of both types of stimulated cells with Boc-2 reversed the inhibitory effect of LXA4 back to baseline.

## Discussion

Previous investigations into the pathogenesis of periodontal disease have typically centered on the role of bacterial infection. However, over the past decade, there has been an increasing interest in the inflammatory response that occurs after infection and which gives rise to osteoclastic bone resorption [29]. In view of this, it has been demonstrated that PDLCs, although not commonly regarded as inflammatory cells, might also play a role and are contributing to the differentiation of osteoclasts by their interaction with osteoclast progenitor cells [30–32]. In periodontal disease, bacterial colonization alters the phenotype of PDLCs by activating inflammatory signaling pathways, which promote tissue-destructive responses [7, 33]. Subsequently, osteoclastic differentiation is promoted by cell-cell-mediated interactions between PDLCs and OPCs—a phenomenon facilitated by adhesion molecules (e.g., ICAM) and release of inflammatory factors (i.e., cytokines, chemokines) [29, 34, 35]. Cell-cell crosstalk between PDLCs and OPCs leads to the migration of TRAP-positive OPCs to the bone surface where osteoclastogenesis occurs, resulting in bone resorption [29, 34, 35]. In the current study, we aimed to set up a similar model, yet in an in vitro environment, to test novel types of medication, which can aid in the treatment of periodontitis. Therefore, a coculture system was set up, closely mimicking the in vivo situation described above.

The periodontal microenvironment contains a wide diversity of oral pathogens which play an important role in periodontal health or disease. The periodontal biofilm is heterogenous with approximately 7000 organisms [36, 37]. Although oral pathogenic bacteria, such as *P. gingivalis*, are commonly associated with PD, “non-oral” pathogens (e.g., *E. coli*) have also recently been implicated in the pathogenesis of this disease [38]. Several studies reported on the presence of non-oral bacterial species after microbiological analysis of the subgingival biofilm of PD patients [39, 40]. Analysis of the subgingival biofilm samples from a large number of untreated chronic PD patients revealed elevated levels of *E. coli* compared with non-PD subjects [39]. Souto and co-workers revealed a positive correlation with clinical signs of PD with bacterial pathogens, including *E. coli*, *Staphylococcus aureus*, *and Pseudomonas aeruginosa* [40]. In primary PDLCs, an inflammatory phenotype was induced by adding bacterial *E. coli* LPS, which in term should trigger osteoclast formation by inducing RANKL expression in osteoblasts and other stimulatory factors (IL-1, prostaglandins, TNFα) leading up to bone resorption [41]. While oral pathogens are more pathologically relevant bacterial source, *E. coli*-derived LPS is more effective at inducing bone resorption in vitro because it is a strong inflammatory agonist for toll-like receptors (TLRs) [42–44] [45]. Conversely, *P. gingivalis* LPS induces a significantly lower expression of inflammatory cytokines (e.g., IL-6) compared with *E. coli* LPS [46]. Considering PDLCs produce a little inflammatory profile, *E. coli* LPS is commonly used to trigger an inflammatory immune response [47]. The subgingival biofilm of PD patients is complex with higher proportion of periodontal pathogens, including lesser known ones, and knowing the effect of different microorganisms is fundamental to our understanding of the complex mechanisms involved in this multifactorial disease.

The mechanism of LPS-induced osteoclastogenesis can be explained as follows. Under inflammatory conditions, LPS-stimulated PDLCs select and attract osteoclast precursors to fuse into osteoclasts by upregulating the expression of osteoclastogenesis-stimulating molecules [5]. During this interaction, LPS binds to TLR4 receptors on PDLCs and stimulates the expression of pro-inflammatory mediators, TNFα, IL-1, and PGE_2_, which play a crucial role in maturation of OPCs and bone resorption [47]. It is important to note that LPS does not promote osteoclast differentiation in the absence of osteoblasts/stromal cells (e.g., PDLCs) [48–50]. Thus, LPS-promoted osteoclastogenesis can be attributed to the direct cell-cell interaction between PDLCs and RAW cells and not from direct interaction of LPS [9, 13]. As a control for our coculture, we also stimulated osteoclast formation artificially, as is commonly done in literature, by the addition of RANKL. The similar results between RANKL stimulation, vs. the effects obtained by LPS/PDLC stimulation, indeed make it plausible that the LPS/PDLC route is a valid representation of the actual situation. Moreover, the subsequent experiments with the CaP coating proved that the RAW cells upon such stimulation became actively matrix-degrading osteoclasts.

After setting up and validating the coculture system with the clinical situation, a suitable drug strategy was selected, for which we focused on the use of SPMs. Among the SPMs released during inflammation, LXs are key to the resolution of inflammation [15, 51]. LXA4 evokes several important protective responses in vivo, including inhibition of neutrophil recruitment, activation, and chemotaxis, via inhibition of several downstream pathways, such as NF-κB, and activator protein-1 (AP-1) [17, 52–57]. Moreover, LXA4 exerts a potent anti-inflammatory action by modulating leukocyte activity and promoting phagocytosis of apoptotic cells [58]. The binding of LXA4 to its receptor (FPR2/ALXR) interferes with osteoclastogenesis by suppressing the expression of inflammatory mediators (e.g., IL-1β and IL-6) via inhibition of multiple signaling pathways, including receptor activator of nuclear factor-κB (NF-κB) [59]. Inhibition of NF-κB suppresses the expression of pro-inflammatory cytokines, which counteracts inflammation-induced bone resorption [60]. The role of LXA4 in inhibition and function of osteoclasts has been recently described in the literature [17]. However, the role of LXA4 as a modulator of cell-cell-mediated osteoclastogenesis remains elusive [61]. Given the lack of studies regarding the role of LXA4 in cell-cell-mediated osteoclast differentiation and function and considering the importance of PDLCs in inflammation-induced osteoclastogenesis, we investigated the effect of LXA4 on osteoclastogenesis promoted by LPS/PDLC challenge, as model system. It was hypothesized that (1) LXA4 would inhibit osteoclast differentiation, and (2) this effect could be reversed by the receptor antagonist, Boc-2. Our data confirmed that both of these hypotheses were tested true.

In the experiments, osteoclastogenesis was analyzed using several complimentary morphological, molecular, and functional assays designed to test all aspects of osteoclast formation, activity, and function. Finally, we performed RT-PCR to fully investigate the mechanism underlying the inhibition of osteoclastogenesis. The results from these studies indicated that RANKL stimulation causes an upregulation in osteoclast differentiation from OPCs. Additionally, osteoclast formation was upregulated during cell-cell contact between PDL and RAW cells when conditioned medium containing bacterial LPS was used. The in vitro experiments demonstrated that PDLCs adapt to bacterial stimuli by upregulating the expression of osteoclastogenesis-stimulating genes, resulting in the release of pro-inflammatory mediators (i.e., cytokines and chemokines) that enhance osteoclast activity and function. Comparison of our results with literature corroborates with most of these effects. For instance, Kanzaki et al. (2001) showed that PDLCs cocultured with peripheral blood monocular cells (PMBCs) exhibited significantly more resorption pits than PMBCs cultured alone [62]. Furthermore, Bloemen et al. (2010) and Burger & Dayer (2002) showed direct that cell-cell contact increased synergistically the expression of osteoclastogenesis genes in vitro [9, 63]. Similar effects were further demonstrated in vivo by Kim et al. (2005), who reported about the formation of osteoclasts, independent of RANKL signaling pathway, in response to stimulation with inflammatory mediators (TNFα and IL-1β) [64]. Considering our results in comparison with the available literature justifies the conclusion that PDLCs contribute to enhanced osteoclast formation in periodontal disease. Evidently, PDLCs can play an important role as a drug target when aiming to maintain hemostasis in the periodontium [5].

Furthermore, the results of the current study showed inhibition osteoclastogenesis in response to inclusion of LXA4 in the differentiation medium. The reduction of osteoclast formation implied a strong protective role of SPMs against inflammation-induced bone resorption, especially as it could be reversed by the addition of a specific inhibitor. Not only the number but also the function of osteoclasts could effectively be modulated; i.e., the decrease in osteoclastogenesis-associated genes was correlated with absence of resorption pits on the CaP-coated substrates. Apparently, LXA4 carries anti-inflammatory capacity after an in vitro encounter with bacterial LPS. In agreement with our data, Liu et al. (2017) showed that LXA4 treatment reduced osteoclast formation in RANKL-stimulated RAW cells [17]. Combination of our data and the literature information confirms again that that both our initial hypotheses were true.

The study has potential limitations. TRAP activity, function, and osteoclast-specific gene expression was notably lower for LPS/PDLC compared with RANKL-stimulated RAW cells. There are several explanations for this effect. Firstly, LPS may negatively affect osteoclast formation by promoting the expression of pro-inflammatory mediators (TNFα and nitric oxide), which may negatively affect cell viability (even at low concentrations) [65–67]. Secondly, RANKL is a strong inducer of osteoclast formation [68, 69], while LPS induces formation of osteoclasts independent of the RANKL pathway by activating pattern recognition receptors, such as TLR4, which can affect activation and survival of osteoclasts [5, 70]. Still, the RANKL stimulation should be considered as an artificial control, whereas the PDLCs route might be more physiologically relevant. Furthermore, murine RAW264.7 monocytes were integrated into the cell culture system because there is currently a lack of a reliable osteoclast model using a human cell line [71]. While the human monocytic leukemia cell line (THP-1) would have presented a more clinically relevant model, these cells are unable to consistently develop into multinucleated osteoclasts and are also far less responsive to LPS [72, 73]. Therefore, it can be challenging to extrapolate the data obtained using this human cell line. On the contrary, murine RAW264.7 cells produce a more robust inflammatory response when challenged with bacterial LPS and have been extensively used to carry out in vitro screens for immunomodulators [74].

## Conclusion

In conclusion, our findings demonstrate the importance of cell-cell signaling between PDLCs and osteoclast precursors in inflammation-induced osteoclastic differentiation. In addition, our data validate the inhibitory role of LXA4 in this process. It is important to note that the current data are the result of in vitro study and might not reflect the clinical in vivo situation. Hence, in vivo (pre)clinical experiments are needed to fully validate whether LXA4 can indeed inhibit osteoclastogenesis and prevent inflammation-induced bone resorption. Nevertheless, the results merit such further investigation and provide important considerations for the implementation of LXA4 in periodontal therapy.

## References

[1] Terrizzi AR, Fernandez-Solari J, Lee CM, Bozzini C, Mandalunis PM, Elverdin JC, Conti MI, Martínez MP (2013). Alveolar bone loss associated to periodontal disease in lead intoxicated rats under environmental hypoxia. Arch Oral Biol.

[2] Hienz SA, Paliwal S, Ivanovski S (2015). Mechanisms of bone resorption in periodontitis %J Journal of Immunology. Research..

[3] Miyazaki T, Miyauchi S, Anada T, Imaizumi H, Suzuki O (2011). Evaluation of osteoclastic resorption activity using calcium phosphate coating combined with labeled polyanion. Anal Biochem.

[4] Takahashi N, Martin TJ, Suda T (1992). Modulation of osteoclast differentiation. Endocr Rev.

[5] Sokos D, Everts V, de Vries TJ (2015). Role of periodontal ligament fibroblasts in osteoclastogenesis: a review. J Periodontal Res.

[6] Beertsen W, McCulloch CAG, Sodek J (1997). The periodontal ligament: a unique, multifunctional connective tissue. Periodontology 2000.

[7] El-Awady AR, Messer RL, Gamal AY, Sharawy MM, Wenger KH, Lapp CA (2010). Periodontal ligament fibroblasts sustain destructive immune modulators of chronic periodontitis. J Periodontol.

[8] Udagawa N, Takahashi N, Jimi E, Matsuzaki K, Tsurukai T, Itoh K, Nakagawa N, Yasuda H, Goto M, Tsuda E, Higashio K, Gillespie MT, Martin TJ, Suda T (1999). Osteoblasts/stromal cells stimulate osteoclast activation through expression of osteoclast differentiation factor/RANKL but not macrophage colony-stimulating factor: receptor activator of NF-kappa B ligand. Bone.

[9] Bloemen V, Schoenmaker T, de Vries TJ, Everts V (2010). Direct cell-cell contact between periodontal ligament fibroblasts and osteoclast precursors synergistically increases the expression of genes related to osteoclastogenesis. J Cell Physiol.

[10] de Vries TJ, Schoenmaker T, Wattanaroonwong N, van den Hoonaard M, Nieuwenhuijse A, Beertsen W, Everts V (2006). Gingival fibroblasts are better at inhibiting osteoclast formation than periodontal ligament fibroblasts. J Cell Biochem.

[11] Wattanaroonwong N, Schoenmaker T, de Vries TJ, Everts V (2011). Oestrogen inhibits osteoclast formation induced by periodontal ligament fibroblasts. Arch Oral Biol.

[12] Hormdee D, Nagasawa T, Kiji M, Yashiro R, Kobayashi H, Koshy G, Noguchi K, Nitta H, Ishikawa I (2005). Protein kinase-A-dependent osteoprotegerin production on interleukin-1 stimulation in human gingival fibroblasts is distinct from periodontal ligament fibroblasts. Clin Exp Immunol.

[13] Bloemen V, Schoenmaker T, de Vries TJ, Everts V (2011). IL-1beta favors osteoclastogenesis via supporting human periodontal ligament fibroblasts. J Cell Biochem.

[14] Buckley CD, Pilling D, Lord JM, Akbar AN, Scheel-Toellner D, Salmon M (2001). Fibroblasts regulate the switch from acute resolving to chronic persistent inflammation. Trends Immunol.

[15] Van Dyke TE, Serhan CN (2003). Resolution of inflammation: a new paradigm for the pathogenesis of periodontal diseases. J Dent Res.

[16] Gaudin A, Tolar M, Peters OA (2018). Lipoxin A4 attenuates the inflammatory response in stem cells of the apical papilla via ALX/FPR2. Sci Rep.

[17] Liu C, Guan H, Cai C, Li F, Xiao J (2017). Lipoxin A4 suppresses osteoclastogenesis in RAW264.7 cells and prevents ovariectomy-induced bone loss. Exp Cell Res.

[18] Hachicha M, Pouliot M, Petasis NA, Serhan CN (1999). Lipoxin (LX)A(4) and aspirin-triggered 15-epi-LXA(4) inhibit tumor necrosis factor 1α–initiated neutrophil responses and trafficking: regulators of a cytokine–chemokine axis. J Exp Med.

[19] Mustafa M, Zarrough A, Bolstad AI, Lygre H, Mustafa K, Hasturk H, Serhan C, Kantarci A, Van Dyke TE (2013). Resolvin D1 protects periodontal ligament. Am J Physiol Cell Physiol.

[20] Sodin-Semrl S, Taddeo B, Tseng D, Varga J, Fiore S (2000). Lipoxin A4 inhibits IL-1 beta-induced IL-6, IL-8, and matrix metalloproteinase-3 production in human synovial fibroblasts and enhances synthesis of tissue inhibitors of metalloproteinases. J Immunol (Baltimore, Md : 1950).

[21] Serhan CN, Sheppard KA (1990). Lipoxin formation during human neutrophil-platelet interactions. Evidence for the transformation of leukotriene A4 by platelet 12-lipoxygenase in vitro. J Clin Invest.

[22] Jimi E, Aoki K, Saito H, D’Acquisto F, May MJ, Nakamura I, Sudo T, Kojima T, Okamoto F, Fukushima H, Okabe K, Ohya K, Ghosh S (2004). Selective inhibition of NF-kappa B blocks osteoclastogenesis and prevents inflammatory bone destruction in vivo. Nat Med.

[23] Serhan CN, Jain A, Marleau S, Clish C, Kantarci A, Behbehani B, Colgan SP, Stahl GL, Merched A, Petasis NA, Chan L, Van Dyke TE (2003). Reduced inflammation and tissue damage in transgenic rabbits overexpressing 15-lipoxygenase and endogenous anti-inflammatory lipid mediators. J Immunol (Baltimore, Md : 1950).

[24] Lee NK, Choi YG, Baik JY, Han SY, D-w J, Bae YS, Kim N, Lee SY (2005). A crucial role for reactive oxygen species in RANKL-induced osteoclast differentiation. Blood.

[25] Patntirapong S, Habibovic P, Hauschka PV (2009). Effects of soluble cobalt and cobalt incorporated into calcium phosphate layers on osteoclast differentiation and activation. Biomaterials.

[26] Maria SM, Prukner C, Sheikh Z, Mueller F, Barralet JE, Komarova SV (2014). Reproducible quantification of osteoclastic activity: characterization of a biomimetic calcium phosphate assay. J Biomed Mater Res B Appl Biomater.

[27] Tran Hle B, Doan VN, Le HT, Ngo LT (2014). Various methods for isolation of multipotent human periodontal ligament cells for regenerative medicine. In Vitro Cell Dev Biol Anim.

[28] Ma M-G (2012) Hierarchically nanostructured hydroxyapatite: hydrothermal synthesis, morphology control, growth mechanism, and biological activity. 7. 10.2147/IJN.S29884 10.2147/IJN.S29884 PMC3356187 22619527

[29] Cochran DL (2008) Inflammation and bone loss in periodontal disease. 79(8S):1569–1576. 10.1902/jop.2008.080233 10.1902/jop.2008.080233 18673012

[30] Page RC, Kornman KS (1997). The pathogenesis of human periodontitis: an introduction. Periodontology 2000.

[31] Offenbacher S (1996). Periodontal diseases: pathogenesis. Ann Periodontol.

[32] Taubman MA, Kawai T, Han X (2007). The new concept of periodontal disease pathogenesis requires new and novel therapeutic strategies. J Clin Periodontol.

[33] Scheres N, Laine ML, de Vries TJ, Everts V, van Winkelhoff AJ (2010). Gingival and periodontal ligament fibroblasts differ in their inflammatory response to viable Porphyromonas gingivalis. J Periodontal Res.

[34] Graves DT, Cochran D (2003). The contribution of interleukin-1 and tumor necrosis factor to periodontal tissue destruction. J Periodontol.

[35] Lerner UH (2006). Inflammation-induced bone remodeling in periodontal disease and the influence of post-menopausal osteoporosis. J Dent Res.

[36] Arora N, Mishra A, Chugh S (2014). Microbial role in periodontitis: have we reached the top? Some unsung bacteria other than red complex. J Indian Soc Periodontol.

[37] Aas JA, Paster BJ, Stokes LN, Olsen I, Dewhirst FE (2005). Defining the normal bacterial flora of the oral cavity. J Clin Microbiol.

[38] Teles R, Teles F, Frias-Lopez J, Paster B (2000). Haffajee A (2013) Lessons learned and unlearned in periodontal microbiology. Periodontol.

[39] Colombo AP, Teles RP, Torres MC, Souto R, Rosalém WJ, Mendes MC, Uzeda M (2002). Subgingival microbiota of Brazilian subjects with untreated chronic periodontitis. J Periodontol.

[40] Souto R, Andrade AFB, Uzeda M, Colombo APV (2006). Prevalence of “non-oral” pathogenic bacteria in subgingival biofilm of subjects with chronic periodontitis. Braz J Microbiol.

[41] Kikuchi T, Matsuguchi T, Tsuboi N, Mitani A, Tanaka S, Matsuoka M, Yamamoto G, Hishikawa T, Noguchi T, Yoshikai Y (2001). Gene expression of osteoclast differentiation factor is induced by lipopolysaccharide in mouse osteoblasts via Toll-like receptors. J Immunol (Baltimore, Md : 1950).

[42] Herath TD, Darveau RP, Seneviratne CJ, Wang CY, Wang Y, Jin L (2013). Tetra- and penta-acylated lipid A structures of Porphyromonas gingivalis LPS differentially activate TLR4-mediated NF-κB signal transduction cascade and immuno-inflammatory response in human gingival fibroblasts. PLoS One.

[43] Chiang N, Fredman G, Bäckhed F, Oh SF, Vickery T, Schmidt BA, Serhan CN (2012). Infection regulates pro-resolving mediators that lower antibiotic requirements. Nature.

[44] Reddi K, Meghji S, Nair SP, Arnett TR, Miller AD, Preuss M, Wilson M, Henderson B, Hill P (1998). The Escherichia coli chaperonin 60 (groEL) is a potent stimulator of osteoclast formation. J Bone Miner Res.

[45] Herath TD, Darveau RP, Seneviratne CJ, Wang CY, Wang Y, Jin L (2013). Tetra- and penta-acylated lipid A structures of Porphyromonas gingivalis LPS differentially activate TLR4-mediated NF-kappaB signal transduction cascade and immuno-inflammatory response in human gingival fibroblasts. PLoS One.

[46] Andrukhov O, Ertlschweiger S, Moritz A, Bantleon H-P, Rausch-Fan X (2013) Different effects of P. gingivalis LPS and E. coli LPS on the expression of interleukin-6 in human gingival fibroblasts. Acta Odontol Scand 72. 10.3109/00016357.2013.834535 10.3109/00016357.2013.834535 24255960

[47] Hassan F, Tumurkhuu G, Dagvadorj J, Koide N, Naiki Y, Mori I, Yoshida T, Yokochi T (2007). Bacterial lipopolysaccharide induces osteoclast formation in RAW 264.7 macrophage cells. Biochem Biophys Res Commun.

[48] Xu J, Zhao Y, Aisa HA (2017). Anti-inflammatory effect of pomegranate flower in lipopolysaccharide (LPS)-stimulated RAW264.7 macrophages. Pharm Biol.

[49] Dong J, Li J, Cui L, Wang Y, Lin J, Qu Y, Wang H (2018). Cortisol modulates inflammatory responses in LPS-stimulated RAW264.7 cells via the NF-kappaB and MAPK pathways. BMC Vet Res.

[50] Zou W, Bar-Shavit Z (2002). Dual modulation of osteoclast differentiation by lipopolysaccharide. J Bone Min Res.

[51] Van Dyke TE (2011). Proresolving lipid mediators: potential for prevention and treatment of periodontitis. J Clin Periodontol.

[52] Chiang N, Serhan CN, Dahlen SE, Drazen JM, Hay DW, Rovati GE, Shimizu T, Yokomizo T, Brink C (2006). The lipoxin receptor ALX: potent ligand-specific and stereoselective actions in vivo. Pharmacol Rev.

[53] Chang J, Wang Z, Tang E, Fan Z, McCauley L, Franceschi R, Guan K, Krebsbach PH, Wang C-Y (2009). Inhibition of osteoblastic bone formation by nuclear factor-κB. Nat Med.

[54] Jimi E, Aoki K, Saito H, D’Acquisto F, May MJ, Nakamura I, Sudo T, Kojima T, Okamoto F, Fukushima H, Okabe K, Ohya K, Ghosh S (2004). Selective inhibition of NF-κB blocks osteoclastogenesis and prevents inflammatory bone destruction in vivo. Nat Med.

[55] Serhan CN, Chiang N, Dalli J (2015). The resolution code of acute inflammation: novel pro-resolving lipid mediators in resolution. Semin Immunol.

[56] Serhan CN, Chiang N, Dalli J, Levy BD (2014). Lipid mediators in the resolution of inflammation. Cold Spring Harb Perspect Biol.

[57] Chiang N, Serhan CN (2017). Structural elucidation and physiologic functions of specialized pro-resolving mediators and their receptors. Mol Asp Med.

[58] Godson C, Mitchell S, Harvey K, Petasis NA, Hogg N, Brady HR (2000). Cutting edge: lipoxins rapidly stimulate nonphlogistic phagocytosis of apoptotic neutrophils by monocyte-derived macrophages. J Immunol (Baltimore, Md : 1950).

[59] Jimi E, Aoki K, Saito H, D’Acquisto F, May M, Nakamura I, Sudo T, Kojima T, Okamoto F, Hidefumi F, Okabe K, Ohya K, Ghosh S (2004). Selective inhibition of NF-??B blocks osteoclastogenesis and prevents inflammatory bone destruction in vivo. Nat Med.

[60] Abu-Amer Y (2013). NF-κB signaling and bone resorption. Osteoporos Int.

[61] Prieto P, Cuenca J, Traves PG, Fernandez-Velasco M, Martin-Sanz P, Bosca L (2010). Lipoxin A4 impairment of apoptotic signaling in macrophages: implication of the PI3K/Akt and the ERK/Nrf-2 defense pathways. Cell Death Differ.

[62] Kanzaki H, Chiba M, Shimizu Y, Mitani H (2001). Dual regulation of osteoclast differentiation by periodontal ligament cells through RANKL stimulation and OPG inhibition. J Dent Res.

[63] Burger D, Dayer J-M (2002). The role of human T-lymphocyte-monocyte contact in inflammation and tissue destruction. Arthritis Res 4 Suppl.

[64] Kim N, Kadono Y, Takami M, Lee J, Lee S-H, Okada F, Kim JH, Kobayashi T, Odgren PR, Nakano H, Yeh W-C, Lee S-K, Lorenzo JA, Choi Y (2005). Osteoclast differentiation independent of the TRANCE–RANK–TRAF6 axis. J Exp Med.

[65] Aldridge C, Razzak A, Babcock TA, Helton WS, Espat NJ (2008). Lipopolysaccharide-stimulated RAW 264.7 macrophage inducible nitric oxide synthase and nitric oxide production is decreased by an omega-3 fatty acid lipid emulsion. J Surg Res.

[66] MacMicking J, Xie Q-w, Nathan C (1997). Nitric oxide and macrophage function. Annu Rev Immunol.

[67] Guzik TJ, Korbut R, Adamek-Guzik T (2003). Nitric oxide and superoxide in inflammation and immune regulation. J Physiol Pharmacol.

[68] Xing L, Schwarz EM, Boyce BF (2005) Osteoclast precursors, RANKL/RANK, and immunology. 208(1):19–29. 10.1111/j.0105-2896.2005.00336.x 10.1111/j.0105-2896.2005.00336.x 16313338

[69] Takayanagi H, Kim S, Taniguchi T (2002). Signaling crosstalk between RANKL and interferons in osteoclast differentiation. Arthritis Res Ther.

[70] Du A, Zhao S, Wan L, Liu T, Peng Z, Zhou Z, Liao Z, Fang H (2016) MicroRNA expression profile of human periodontal ligament cells under the influence of Porphyromonas gingivalis LPS. 20(7):1329–1338. 10.1111/jcmm.12819 10.1111/jcmm.12819 PMC4929301 26987780

[71] Li ZH, Si Y, Xu G, Chen XM, Xiong H, Lai L, Zheng YQ, Zhang ZG (2017). High-dose PMA with RANKL and MCSF induces THP-1 cell differentiation into human functional osteoclasts in vitro. Mol Med Rep.

[72] Diana B, Paola I (2016). Model validity in nanoimmunosafety: advantages and disadvantages of in vivo vs in vitro models, and human vs animal models. Curr Bionanotechnol(Discontinued).

[73] Bosshart H, Heinzelmann M (2016). THP-1 cells as a model for human monocytes. Ann Transl Med.

[74] Elisia I, Pae HB, Lam V, Cederberg R, Hofs E, Krystal G (2018). Comparison of RAW264.7, human whole blood and PBMC assays to screen for immunomodulators. J Immunol Methods.

